# Rotablation in Patients with Advanced Renal Insufficiency through End-Stage Renal Disease: Short- and Intermediate-Term Results

**DOI:** 10.1155/2022/7884401

**Published:** 2022-03-10

**Authors:** Wei-Jung Lo, Wei-Jhong Chen, Chih-Hung Lai, Yu-Wei Chen, Chieh-Shou Su, Wei-Chun Chang, Chi-Yan Wang, Tsun-Jui Liu, Kae-Woei Liang, Wen-Lieng Lee

**Affiliations:** ^1^Cardiovascular Center, Taichung Veterans General Hospital, Taichung 407, Taiwan; ^2^Institute of Clinical Medicine, National Yang Ming Chiao Tung University School of Medicine, Taipei 112, Taiwan; ^3^Department of Medicine, National Yang Ming Chiao Tung University School of Medicine, Taipei 112, Taiwan; ^4^Ministry of Health and Welfare Feng-Yuan Hospital, Taichung 420, Taiwan

## Abstract

**Objective:**

Patients with advanced renal insufficiency are at high risk of coronary artery disease (CAD) and complex lesions. Treating complex calcified lesion with rotational atherectomy (RA) in these patients might be associated with higher risks and poorer outcomes. This study was set to evaluate features and outcomes of RA in these patients.

**Method:**

Consecutive patients who received coronary RA from April 2010 to April 2018 were queried from the Cath Lab database. The procedural details, angiography, and clinical information were reviewed in detail.

**Results:**

A total of 411 patients were enrolled and divided into Group A (baseline serum creatinine <5 mg/dl, *n* = 338) and Group B (baseline serum creatinine ≥ 5 mg/dl through ESRD, *n* = 73). Most patients had high-risk features (65.7% of acute coronary syndrome (ACS), 14.1% of ischemic cardiomyopathy, and 5.1% of cardiogenic shock). Group B patients were significantly younger (66.8 ± 11.4 vs. 75.2 ± 10.7 years, *p* < 0.001) and had more RCA and LCX but less LAD treated with RA. No difference was found in lesion location, vessel tortuosity, bifurcation lesions, chronic total occlusion, total lesion length, or total lesion numbers between the two groups. Less patients in Group B obtained completion of RA (95.9% vs 99.1%, *p*=0.037). There was no difference in the incidence of procedural complication or acute contrast-induced nephropathy. Group B patients had more deaths and MACE while in the hospital. The MACE and CV MACE were also higher in Group B patients at 180 days and one year, mostly due to TLR and TVR. Multivariate regression analysis showed that ACS, age, peripheral artery disease (PAD), advanced renal insufficiency, ischemic cardiomyopathy/shock, and high residual SYNTAX score were independent risk factors for in-hospital MACE, whereas ACS, advanced renal insufficiency, ischemic cardiomyopathy/shock, triple-vessel disease, and PAD independently predicted MACE at 6 months.

**Conclusions:**

Rotablation is feasible, safe, and could be carried out with very high success rate in very-high-risk patients with advanced renal dysfunction through ESRD without an increase in procedural complication.

## 1. Introduction

Coronary artery calcifications are common in coronary artery disease (CAD), with approximately 38% of lesions calcified as shown in coronary angiography [1]. In addition to the increasing incidence of CAD, patients with renal impairment have an increased risk of severe coronary calcifications and it could reach 60% in patients with chronic kidney disease (CKD) [2, 3]. Percutaneous coronary intervention (PCI) for calcified coronary lesions is associated with remarkably worse outcomes compared with noncalcified lesions, even in the drug-eluting stent (DES) era [4, 5]. Besides, severely calcified lesions are difficult to be crossed or dilated with balloon or stent and are associated with an increased risk of restenosis and target lesion revascularization (TLR) [6–8]. Patients with advanced renal insufficiency or ESRD are one of the most challenging populations with respect to technical difficulty and potential risk of complications in PCI [9]. The incidence of major adverse cardiovascular events (MACEs), mortality, and target vessel revascularization (TVR) was significantly higher in this population [10].

To mitigate the poor results associated with coronary calcifications, lesion preparation in PCI is necessary to facilitate balloon and stent delivery, and full vessel expansion. Rotablation atherectomy (RA) is one of the standard strategies for device-uncrossable or device-undilatable calcified lesions. In recent years, complex and high-risk coronary interventions (CHIPs) have attracted much attention because many such patients gain benefits from revascularization while demanding particular cares and intervention techniques during procedure [11, 12]. Using RA accounted for 1–3% of PCI in the UK, Europe, and the USA [13].

There were very limited studies investigating the impact of renal insufficiency on the outcomes of coronary RA in the literature [14, 15], especially in very-high-risk patients. This retrospective study was set to evaluate the clinical features, procedural details, and immediate/intermediate results of RA in patients with advanced renal insufficiency through ESRD.

## 2. Methods

### 2.1. Patient Population

This was a retrospective study. Consecutive patients who received RA therapy for coronary lesions from April 2010 to April 2018 at our Cath Labs were interrogated from the Cath Lab database and identified by manual inspection. The indications for PCI and RA, procedural details, and complications at the time of index PCI were retrieved. The admission CAD diagnosis for coronary intervention was divided into stable angina, unstable angina, NSTEMI, STEMI, and ischemic cardiomyopathy. The first four diagnoses were made according to the commonly used ESC guidelines [16, 17]. The diagnosis of ischemic CM was made if the patients presented with no chest pain but clinical heart failure or acute pulmonary edema without or without respiratory failure. Patients with the above diagnoses might also simultaneously present with cardiogenic shock except those with stable angina or limited unstable angina. Cardiogenic shock was defined as systolic blood pressure lower than 90 mmHg after appropriate fluid supplement together with clinical or laboratory evidence of hypoperfusion, including those who remained in a similar or worse status despite high-dose vasopressor support greater than 0.5 *μ*g/kg/min of norepinephrine or equivalent.

The computerized electronic medical chart records of each patient were reviewed in detail and relevant clinical information, and biochemical findings at the time of hospitalization were retrieved and recorded in the case record form. Patients were stratified into two groups based on baseline renal function: serum creatinine of <5 mg/dl (Group A) or ≥5 mg/dl through ESRD (Group B). Though GFR is currently the standard to measure renal function, the use of GFR or eGFR rather than serum creatinine is most important in early detection and early prevention of renal disease, when the serum creatinine level could not reflect real renal function. However, in patients with advanced renal dysfunction, the rise in serum creatinine was steep and reflect real renal function [18]. When the serum creatinine is 400–500 mmol/L (4.5–5.5 mg/dl), any more decline in GFR is associated with dramatic rise in serum creatinine and serum creatinine of 5 mg/dl approximates CKD stage V. On the other hand, the physician-familiar and long-time used maximal allowable contrast dose (MACD) uses serum creatinine but not GFR(eGFR) in the simple calculation of contrast dose for intervention (5 times body weight (in kg) divided by serum creatinine) [19]. As this study aimed to investigate the feasibility, safety, and efficacy of rotablation in patients with advanced renal insufficiency, we decided to use the simple and straightforward serum creatinine cutoff value of 5 mg/dl to dichotomize the patients.

Acute contrast-induced nephropathy (CIN) following the rotablation procedure was defined traditionally as rise in serum creatinine of >0.5 mg/dl or >25% in 48 hours in non-dialysis patients. For patients under regular hemodialysis at the baseline, the detection of CIN was not possible and not intended.

### 2.2. Angiographic Characterization and Measurements

Workstation with dedicated software (Rubo DICOM Viewer, Version 2.0, Build 170828, Rubo Medical Imaging, Aerdenhout, The Netherlands) was used to review the coronary angiograms and make quantitative measurements. The SYNTAX scores before and after PCI were calculated using the standard calculator software on the website. CAD vessel numbers were defined as the number of the three major coronary vessels with stenosis ≥70% in diameter. Severe coronary artery calcification was defined as apparent abluminal radio-opacity on two sides of the vascular walls appearing in two different projections on the cine without cardiac movement and before the injection of contrast medium.

All PCIs were performed by certified interventional cardiologists in accordance with the standard practice at our Cath Lab. Patients were pretreated with a standard dose of aspirin and clopidogrel (or ticagrelor). Calcium channel blocker and nitrate were also used to prevent coronary artery spasm. Heparin was administered to maintain an activated clotting time (ACT) of ≥300 seconds during procedure. The decision to do RA was determined by standard practice and also at the discretion of the operator. Prior to RA, a 0.009-inch floppy RotaWire^TM^ was advanced through the lesion using the wire exchange technique. A bolus of 1,200–1,600 ug of isosorbide dinitrate was given intracoronary prior to the start of RA, during which normal saline mixed with heparin and isosorbide dinitrate was slowly infused. RA was implemented using the Rotablator^TM^ RA system, starting with a 1.25 or 1.5 mm burr at a speed of 180,000–200,000 rpm, and this was often supplemented with a second burr one size bigger. Each burr advance time was less than 20 seconds. For patients who needed side branch (SB) rotablation, the sequence of RA of SB or main vessel (MV) was determined by which vessel was more critically diseased and potentially jeopardized if not treated first and at the discretion of the operator. After the accomplishment of RA, workhorse wire replaced RotaWire^TM^ using the same wire exchange technique and the procedure proceeded with balloon angioplasty with or without stent implantation to achieve optimal angiographic results and minimal residual stenosis. Whenever indicated, glycoprotein IIb/IIIa inhibitor or inotropic was administered. The completion of RA was defined as full debulking of the target lesion without premature termination of RA before proceeding to subsequent treatment. After stent implantation, dual-antiplatelet therapy with aspirin (100 mg/day) and clopidogrel (75 mg/day; or ticagrelor 90 mg twice a day) were continued for at least 12 months in the case of DES or three months in the case of bare-metal stent (BMS) implantation. The above method was used and had been published in our previous works [12, 20, 21].

### 2.3. Clinical Outcomes

The computerized electronic medical chart records of each patient were reviewed in detail, and relevant clinical information (occurrence of death, myocardial infarction, stroke, and coronary revascularization) at different time points (in the hospital, at 30 days, 90 days, 180 days, and 1 year after index PCI) was retrieved and recorded in the case record form. Telephone contacts were made if patients had missed any follow-up sessions for a period over two months since the last visit. In case of patient mortality, the cause of death as stated in the death certificate was retrieved.

The major adverse cardiovascular events (MACEs) were defined as total death, myocardial infarction, stroke, and coronary revascularization. The cardiovascular major adverse cardiac events (CV MACEs) were defined as cardiovascular death, myocardial infarction, stroke, and coronary revascularization. TLR was defined as performing any procedure for lumen narrowing, which was attributed to restenosis of the index-treated lesion. TVR refers to repeated PCI for a lesion in another segment of the vessel not treated at the index procedure or TLR occurred. This study protocol was approved by the Institutional Review Board for Human Research of Taichung Veterans General Hospital, Taiwan.

### 2.4. Statistical Analysis

Categorical data were expressed as number and frequency. Continuous variables were presented as mean ± standard deviation. Differences in categorical data were compared using the chi-square test, and differences in continuous variables were measured by the unpaired Student's t-test. Multivariate binary logistic regression analyses were used to identify independent predicting factors for MACE or CV MACE at different follow-up periods. All statistical analyses were presented with the IBM SPSS Statistical Software for Microsoft Windows, Version 26.0 (IBM Corp., New York, USA). Two-tailed *p* values below 0.05 were considered statistically significant.

## 3. Results

### 3.1. Baseline Characteristics of Patients

A total of 411 patients, 269 males and 142 females, with a mean age of 73.8 ± 11.3 years were enrolled. Most patients had high-risk features (65.7% of acute coronary syndrome (ACS), 14.1% of ischemic cardiomyopathy, and 5.1% of cardiogenic shock), and only 20.2% of patients had stable angina. 73.5% of patients presented with hypertension, 58.6% with diabetes, and 10.7% with peripheral artery disease (PAD).

Among them, 338 patients (82.2%, 221 males and 117 females) had serum creatinine of <5 mg/dl (Group A), whereas another 73 patients (17.8%, 48 males and 25 females) had serum creatinine of ≥5 mg/dl through ESRD (advanced renal insufficiency, Group B). The baseline characteristics for all patients are presented in Table 1. Patients in Group B were significantly younger (66.8 ± 11.4 vs. 75.2 ± 10.7 years, *p* < 0.001) and had lower hemoglobin (10.1 ± 2.0 vs 11.6 ± 2.1 mg/dl, *p* < 0.001) and LDL cholesterol (76.6 ± 23.8 vs 87.1 ± 29.0 mg/dl, *p*=0.014). There was no statistically significant difference in CAD vessel numbers or LVEF between the two groups. The mean CKD-EPI eGFR in Group B was only 6.5 ± 1.9 ml/min/1.73 m^2^ and significantly lowered than that of Group A patients (57.8 ± 28.2.2 ml/min/1.73 m^2^, *p* < 0.001).

### 3.2. Lesion Characteristics and Rotablation Procedural Details

Lesion characteristics and procedural details are presented in Table 2. Overall, type C lesions were seen in most of the patients (90.8%). The majority of rotablation was done for main vessel only (86.9%) and for single vessel (82.5%). Rotablation for LM lesions was done in 8.8% of these individuals. Burr size of 1.5 mm or above (82.7%) and DES (68.4%) were more often used.

Group B patients had more RCA (27.4% vs 16.9%) and LCX (13.7% vs 7.7%) but less LAD (39.7% vs 58.0%) treated with RA. No differences were seen in rotablation for two or three vessels between these two groups. Femoral access (90.4% vs 61.8%, *p* < 0.001) and 7/8 F sheaths (79.5% vs 66.3%, *p*=0.026) were used more frequently in Group B patients. No significant difference was found in lesion location, vessel tortuosity, bifurcation lesions, CTO, total lesion length, or total lesion numbers between the two groups. Less patients in Group B obtained completion of RA (95.9% vs 99.1%, *p*=0.037). There was no difference in stent numbers or size, total stent length, baseline/post-PCI SYNTAX score, total procedure time, fluoroscopic time, contrast dose, or use of hemodynamic support during PCI between these two groups.

### 3.3. Procedural Outcomes

The procedural outcomes and incidence of acute CIN are presented in Table 3. There was no significant difference in the incidence of acute slow/no flow, wire transection, vessel perforation, acute heart failure, ventricular arrhythmia, acute CIN, or use of IIb/IIIa inhibitor between Group A and Group B. However, a higher incidence of procedural cardiogenic shock was noted in Group A (13.9% vs. 4.1%, *p*=0.020). No patient demanded emergent CABG or died during procedure.

### 3.4. In-Hospital, Short-Term, and Intermediate-Term Clinical Outcomes

In-hospital, 30-day, 90-day, 180-day, and one-year clinical outcomes are presented in Table 4. Group B patients had higher deaths (12.3% vs 5.3%, *p*=0.029) and MACE (13.7% vs 6.2%, *p*=0.028) while in the hospital but not at one or three months. The MACE and CV MACE were also higher in Group B patients at 180 days (37.0% vs 21.6%, *p*=0.006, and 28.8% vs 16.0%, *p*=0.011, respectively) and at one year (47.9% vs 28.4%, *p*=0.001, and 37.0% vs 19.2%, *p*=0.001, respectively), mostly due to higher TLR (17.8% vs 6.2%, *p*=0.001, and 23.3% vs 9.2%, *p*=0.001, respectively) and TVR (20.5% vs 7.7%, *p*=0.001, and 27.4% vs 10.9%, *p*=0.001, respectively) at the time points and also higher fatal MI at 12 months (5.5% vs 1.2%, *p*=0.016). There was no difference in CV death, nonfatal MI, stent thrombosis, or stroke between these two groups at any time point.

### 3.5. Predicting Factors for Clinical Outcomes

Multivariate binary logistic regression analysis showed that the diagnosis of ACS, age, PAD, advanced renal insufficiency, ischemic cardiomyopathy/shock, and high residual SYNTAX score were independent risk factors for in-hospital MACE. Furthermore, diagnosis of ACS, advanced renal insufficiency, ischemic cardiomyopathy/shock, triple-vessel disease, and PAD independently predicted MACE at 6 months (Table 5).

## 4. Discussion

In brief, our study found that patients with advanced renal insufficiency through ESRD undergone RA had lower hemoglobin, LDL cholesterol, and very-high-risk clinical features, but were younger than their counterparts with better renal function. Secondly, RA could be safely performed in these patients without an increase in periprocedural complication despite more RCA and LCX rotablation and use of larger sheaths via femoral access. Thirdly, there were significantly higher in-hospital MACE and 6- and 12-month MACE/CV MACE in patients with advanced renal insufficiency through ESRD, driven by higher deaths in the hospital but higher TLR and TVR at 6 and 12 months. Finally, the in-hospital MACE of RA patients could be predicted by advanced renal insufficiency per se, age, PAD, ACS presentation, ischemic cardiomyopathy/shock, and higher residual SYNTAX score. On the other hand, the intermediate-term outcome was associated with advanced renal insufficiency per se, PAD, ACS presentation, ischemic cardiomyopathy/shock, and underlying triple-vessel numbers.

Cardiovascular morbidity and mortality in patients with CKD are high, and the presence of CKD worsens outcomes of cardiovascular disease [22]. The prevalence of CAD in ESRD ranges from 25% in young nondiabetic patients to 85% in older ESRD patients with long-term diabetes, where the prevalence of CAD could reach 44.3% in patients with advanced renal insufficiency but no ESRD based on the USRDS survey in 2018 [23]. As the incidence and prevalence of ESRD (and therefore permanent dialysis) in Taiwan rank top in the USRDS survey, both the diagnosis and management of the vast number of CAD in these patients constitute major challenges to not only nephrologists but also cardiologists in this country. Coronary lesions in patients with ESRD are characterized by diffuse diseases with long segmental involvements, long circular/rotating heavy calcification, and chronic total occlusions with the involvement of left main and bifurcations [2, 24]. Patients with CKD also frequently present with complex PAD and other systemic comorbidities, making them a subgroup of patients deserving particular attention before, during, and after coronary interventions [22]. Similar findings were born out in this study as patients with advanced renal insufficiency through ESRD were significantly younger (8.4 years) than those with better renal function, despite similar lesion complexities requiring coronary RA. Most (78.1%) of these patients presented as ACS or ischemic cardiomyopathy, and 85% of these patients had multiple vessel diseases. The total SYNTAX score was also high up to 30.5 at the baseline, arguing for the complexity of the coronary lesions and challenges in PCI.

In recent years, CHIP draws a lot of attention because of challenges during PCI and multiple comorbidities [11]. A lot of CHIP refers to patients with advanced renal insufficiency, who are old in age and frail and have many comorbidities [25, 26]. There were high incidences of hypertension, diabetes, and PAD in our patients with advanced renal insufficiency, who also had lower hemoglobin and depressed LV function. Challenges in PCI for patients with advanced renal insufficiency or ESRD include lesions, which are not uncommonly device-undilatable or device-uncrossable, vessel dissection or rupture if forceful dilatation is used, vessel tortuosity, and difficulty in achieving full stent expansion and apposition [9]. Most of these are caused by heavy calcification [2, 3]. Our patients with advanced renal insufficiency had similar total lesion lengths and numbers to be treated with rotablation as compared with their counterparts. These patients also had more LCX and RCA rotablated in comparison with more LAD debulked in those with better renal function. Despite these disadvantages, rotablation could be completed in an outstanding (95.9%) proportion of our sick patients with poor renal function without increase in procedural complications. Unexpectedly, less periprocedural hypotension or shock occurred in our sick patients.

Patients with advanced renal insufficiency are prone to restenosis following PCI and poorer clinical outcomes, even with the use of DES [10, 27]. In the Euro4C study looking at the contemporary use and outcomes of RA in Europe, the predictors for MACE at one year were female gender, renal failure, ACS at admission, depressed LV function, and significant LM disease [28]. However, in contrast to our study with very high ACS rate, ACS was present (2.3) only in 25.1% of their patients. LV dysfunction, renal insufficiency, cardiogenic shock, and DM were independent risk factors for mortality in the study by Edes et al [29]. The J2T multicenter registry in Japan also showed that age, hemodialysis, multivessel disease, low LVEF, CRP, and use of statins were related to CV mortality in RA patients [30].

There were only limited studies of RA in patients with ESRD or advanced renal insufficiency. A substudy of the J2T in patients with ESRD found that upsizing of burr size, final TIMI 3 flow, lower BNP level, and optimal medications but not others were predicting factors for CV deaths [14]. Again, ACS was presented in only 18% of their patients and they did not report procedural outcomes. Malik FTN et al. studied the safety and efficacy of RA with new-generation DES in patients with CKD [15]. The procedural success was very high (97.5%), and procedural complications were low. However, severe CKD was presented in only 6.5% of patients, in contrast to 17.8% in our study. Their patients were also younger than ours (63.9 ± 8.8 vs 66.8 ± 11.4 years) and with much less ACS (27% vs 78.1%). No comparison in outcomes was made between patients with different degrees of renal insufficiency. Our study might be the first one to look at the safety and efficacy of RA in very-high-risk patients with advanced renal insufficiency through ESRD and compared them with their counterparts with better renal function. We also attempted to find the risk factors for outcomes in these particular groups of patients. In our study, we found that the rotablation in patients with advanced renal dysfunction was associated with increased MACE, particularly total deaths, in the hospital. However, this was not caused by rotablation or procedural complications per se but by comorbidities. After hospital discharge, there was no difference in MACE at one month or three months. Though the MACE and CV MACE at 6 and 12 months were higher in patients with advanced renal dysfunction through ESRD, they were attributed to TLR and TVR. It is interesting to note that our patients with advanced renal dysfunction patients were younger, arguing for primary causes for renal diseases and rapid progression of CAD to advanced calcification necessitating rotablation. The difference in age might be one of the causes that the efficacy and safety of rotablation in patients with advanced renal dysfunction were the same as patients with better renal function in our study.

There are several limitations of this study. Firstly, the retrospective design is subject to all its inherent limitations. Secondly, the study population was heterogeneous in clinical diagnoses and had varying clinical presentations. However, this study did reflect the real-world practice when heavily calcified complex lesions in very-high-risk patients demanded rotablation a priori or in a bail-out manner to complete revascularization and achieve good immediate results. However, the large population does allow us to do multivariate regression analysis to explore the independent risk factors for MACE or CV MACE. Thirdly, the study population spanned over 10 years in which the PCI devices, skills, and experiences improved over time, but disease severity and lesion complexity in PCI also increased as a trade-off. These were hard to control in this study. Again, this study in a larger patient population was meant to explore the feasibility, safety, and efficacy of RA in very-high-risk patients with advanced renal insufficiency in real-world practice. Fourthly, the incidence of acute CIN depends on multiple risk factors and is expected to be higher in patients with higher baseline serum creatinine. Despite the incidence of acute CIN in both groups being similar, the detection of acute CIN in ESRD patients was not possible and might underestimate the true incidence of acute CIN in Group B. Lastly, the cutoff value of renal function was arbitrarily set as serum creatinine of 5 mg/dl. Choosing other cutoff values may affect the study results. However, serum creatinine of 5 mg/dl is a simple and logical surrogate marker of advanced renal insufficiency and impending end-stage renal disease in clinical practice as we mentioned in the Methods. What patients with this stage of renal disease would fate after complex PCI with RA is an intriguing question to answer.

## 5. Conclusions

RA is feasible, safe, and could be carried out with very high success rate but without an increase in procedural complication in very-high-risk patients with advanced renal dysfunction through ESRD. The very short-term clinical results were attributed to the presence of PAD, residual SYNTAX score, ACS, advanced renal insufficiency per se, and ischemic cardiomyopathy/shock. On the other hand, the longer-term results were associated with the presence of PAD, ACS, advanced renal insufficiency, triple-vessel diseases, and ischemic cardiomyopathy/shock. There were higher TLR and TVR in these patients during follow-up, but they were not independent predictors for MACE or CV MACE.

## Figures and Tables

**Table 1:** Demographic data and CAD vessel numbers in rotablation patients.

Variables	Group A	Group B	*p* value
	(CR < 5 mg/dl)	(CR ≥ 5 mg/dl through ESRD)	
	*N* = 338	*N* = 73	
Sex (M/F)	221/117	48/25	0.952
Age (years)	75.2 ± 10.7	66.8 ± 11.4	<**0.001**
Clinical diagnosis (*N*, %)			
Stable angina	67 (19.8%)	16 (21.9%)	0.472
Unstable angina	120 (35.5%)	27 (37.0%)	
NSTEMI	76 (22.5%)	14 (19.2%)	
STEMI	13 (3.8%)	1 (1.4%)	
Ischemic CM	45 (13.3%)	11 (15.1%)	
Cardiogenic shock	17 (5%)	4 (5.5%)	
Hypertension (*N*, %)	249 (73.7%)	53 (72.6%)	0.852
Diabetes (*N*, %)	201 (51.0%)	40 (58.3%)	0.462
PAD (*N*, %)	32 (9.5%)	12 (16.4%)	0.081
Baseline LVEF (%)	45.9 ± 12.5	44.1 ± 13.1	0.335
Laboratory data			
Hemoglobin (mg/dl)	11.6 ± 2.1	10.1 ± 2.0	<**0.001**
BUN (mg/dl)	35.5 ± 74.3	66.3 ± 24.6	**0.013**
CR (mg/dl)	1.6 ± 1.0	8.2 ± 2.3	**<0.001**
CKD-EPI eGFR (ml/min/1.73 m^2^)	57.8 ± 28.2.2	6.5 ± 1.9	**<0.001**
Cholesterol (mg/dl)	150.1 ± 31.9	141.8 ± 32.9	0.078
HDL chol (mg/dl)	45.0 ± 13.7	42.6 ± 13.8	0.264
LDL chol (mg/dl)	87.1 ± 29.0	76.6 ± 23.8	**0.014**
FBS (mg/dl)	151.9 ± 85.8	149.8 ± 93.5	0.874
HbA1c (mg/dl)	6.8 ± 1.4	6.5 ± 1.5	0.318
Total CK (U/L)	199.9 ± 338.3	198.0 ± 289.5	0.967
CK-MB (U/L)	10.6 ± 11.9	14.3 ± 18.8	0.056
Troponin (ng/ml)	3.3 ± 9.2	5.6 ± 14.7	0.130
CAD vessel numbers			
SVD (*N*, %)	79 (23.4%)	11 (15.1%)	0.415
DVD (*N*, %)	94 (27.8%)	17 (23.3%)	
TVD (*N*, %)	107 (31.7%)	35 (47.9%)	
Plus LM (*N*, %)	41 (12.1%)	7 (9.6%)	
Prior CABG (*N*, %)	17 (3.3%)	3 (4.1%)	

CABG, coronary artery bypass grafting; CAD, coronary artery disease; CM, cardiomyopathy; CR, creatinine; DVD, double-vessel disease; ESRD, end-stage renal disease; FBS, fasting blood sugar; LM, left main coronary artery; NSTEMI, non-ST elevation myocardial infarction; PAD, peripheral artery disease; STEMI, ST elevation myocardial infarction; SVD, single-vessel disease; TVD, triple-vessel disease.

**Table 2:** Lesion characteristics and procedural details of rotational atherectomy in patients with CR < 5 mg/dl and CR ≥ 5 mg/dl through ESRD.

Variables	Group A	Group B	*p* value
	(CR < 5 mg/dl) *N* = 338	(CR ≥ 5 mg/dl through ESRD) *N* = 73	
Access site			
Radial (*N*, %)	120 (35.5%)	3 (4.1%)	<**0.001**
Femoral (*N*, %)	209 (61.8%)	66 (90.4%)	
Brachial (*N*, %)	9 (2.7%)	4 (5.5%)	
Guide size			
6F (*N*, %)	114 (33.7%)	15 (20.5%)	**0.026**
7F (*N*, %)	220 (65.1%)	55 (75.3%)	
8F (*N*, %)	4 (1.2%)	3 (4.1%)	
Rotablation vessels			
LM (*N*, %)	1 (0.3%)	0	**0.035**
LAD (*N*, %)	196 (58.0%)	29 (39.7%)	
LCX (*N*, %)	26 (7.7%)	10 (13.7%)	
RCA (*N*, %)	57 (16.9%)	20 (27.4%)	
LM + LAD (*N*, %)	16 (4.7%)	2 (2.7%)	
LM + LCX (*N*, %)	6 (1.8%)	2 (2.7%)	
LM + RCA (*N*, %)	1 (0.3%)	0	
LAD + LCX (*N*, %)	20 (6.0%)	3 (4.1%)	
LAD + RCA (*N*, %)	10 (3.0%)	3 (4.1%)	
LCX + RCA (*N*, %)	1 (0.3%)	0	
LM + LAD + LCX (*N*, %)	1 (0.3%)	3 (4.1%)	
LM + LAD + RCA (*N*, %)	3 (0.9%)	1 (1.4%)	
Rotablation lesions			
Location			
Ostial (*N*, %)	123 (36.4%)	26 (35.6%)	0.914
Proximal (*N*, %)	251 (74.3%)	55 (75.3%)	0.762
Middle (*N*, %)	309 (91.4%)	65 (89.0%)	0.812
Distal (*N*, %)	238 (70.4%)	50 (68.5%)	0.519
Tortuosity (*N*, %)	163 (48.2%)	34 (46.6%)	0.135
Bifurcation (*N*, %)	114 (33.7%)	20 (27.4%)	0.405
Heavy calcification (*N*, %)	337 (99.7%)	72 (98.6%)	0.854
Chronic total occlusion (*N*, %)	46 (13.6%)	9 (12.3%)	0.771
Total lesion length (mm)	44.6 ± 24.3	46.3 ± 25.8	0.584
Total lesion numbers (*N*)	2.4 ± 1.1	2.5 ± 1.3	0.316
ACC/AHA lesion (*N*, %)			
B2	29 (8.6%)	9 (12.3%)	0.482
C	309 (91.4%)	64 (87.7%)	
Main/side branch rotablation (*N*, %)			
Main vessel only	291 (86.1%)	66 (90.4%)	0.565
Main + side branch	31 (9.2%)	4 (5.5%)	
Side branch only	16 (4.7%)	3 (4.1%)	
Maximum burr size			
1.25 mm (*N*, %)	57 (16.9%)	14 (19.2%)	0.217
1.5 mm (*N*, %)	201 (59.5%)	38 (52.1%)	
1.75 mm (*N*, %)	75 (22.2%)	19 (26.0%)	
2.0 mm (*N*, %)	5 (1.5%)	1 (1.4%)	
2.25 mm (*N*, %)	0	1 (1.4%)	
Rotablation completed	335 (99.1%)	70 (95.9%)	**0.037**
Stents (*N*, %)	309 (91.4%)	63 (86.3%)	0.155
BMS (*N*, %)	76 (24.6%)	14 (22.2%)	
DES (*N*, %)	232 (75.1%)	48 (76.2%)	
DEB (*N*, %)	1 (0.3%)	0	
BMS + DES (*N*, %)	0	1 (1.4%)	
Mean stent numbers	1.9 ± 0.9	2.0 ± 0.9	0.498
Mean stent size (mm)	3.0 ± 2.2	2.9 ± 0.4	0.692
Total stent length (mm)	52.1 ± 27.2	54.6 ± 28.0	0.568
SYNTAX score			
Baseline	31.0 ± 14.3	30.5 ± 14.7	0.784
Post-PCI	8.4 ± 10.6	9.1 ± 9.5	0.580
Gain	22.6 ± 11.5	21.3 ± 11.2	0.397
Total procedure time (min)	160 ± 57	163 ± 74	0.726
Total fluoroscopic time (min)	46.6 ± 22.7	50.0 ± 29.7	0.277
Total contrast dose (ml)	194.1 ± 73.4	198.1 ± 66.4	0.695
Hemodynamic support (*N*, %)	55 (16.3%)	8 (11.0%)	0.253

BMS, bare-metal stent; CR, creatinine; DEB, drug-eluting balloon; DES, drug-eluting stent; ESRD, end-stage renal disease; LAD, left anterior descending artery; LCX, left circumflex artery; LM, left main coronary artery; PCI, percutaneous coronary intervention; RCA, right coronary artery.

**Table 3:** Incidence of in-procedural complications and acute contrast-induced nephropathy in patients with CR < 5 mg/dl and CR ≥ 5 mg/dl through ESRD.

Variables	Group A	Group B	*p* value
	(CR < 5 mg/dl through ESRD)	(CR >= 5 mg/dl through ESRD)	
	*N* = 338	*N* = 73	
Acute slow/no flow (*N*, %)	31 (9.2%)	4 (5.5%)	0.305
Wire transection (*N*, %)	1 (0.3%)	0	—
Vessel perforation (*N*, %)	4 (1.2%)	1 (1.4%)	0.895
Acute heart failure (*N*, %)	13 (3.8%)	1 (1.4%)	0.290
Profound/refractory shock	47 (13.9%)	3 (4.1%)	**0.020**
Ventricular arrhythmia (*N*, %)	7 (2.1%)	2 (2.7%)	0.723
Emergent CABG (*N*, %)	0	0	—
Die on table (*N*, %)	0	0	—
Acute CIN (*N*, %)^*∗*^	17 (5.0%)	4 (5.5%)	0.863
Access hematoma (*N*, %)	10 (3.0%)	0	0.136
IIb/IIIa inhibitors (*N*, %)	8 (2.4%)	1 (1.4%)	0.594

CABG, coronary artery bypass grafting; CIN, contrast-induced nephropathy; CR, creatinine; ESRD, end-stage renal disease. ^*∗*^Excluding patients on regular hemodialysis.

**Table 4:** Clinical outcomes of rotational atherectomy in patients with CR < 5 mg/d and CR ≥ 5 mg/dl through ESRD.

Variables	Group A	Group B	*p* value
	(CR < 5 mg/dl)	(CR ≥ 5 mg/dl through ESRD)	
	*N* = 338	*N* = 73	
In-hospital			
MACE (*N*, %)	21 (6.2%)	10 (13.7%)	**0.028**
CV MACE (*N*, %)	17 (5.0%)	5 (6.8%)	0.531
Death (*N*, %)	18 (5.3%)	9 (12.3%)	**0.029**
CV death (*N*, %)	13 (3.8%)	4 (5.5%)	0.525
Fatal MI (*N*, %)	1 (0.3%)	1 (1.4%)	0.232
Nonfatal MI (*N*, %)	3 (0.9%)	0	0.419
Stent thrombosis (*N*, %)	1 (0.3%)	0	0.642
Stroke (*N*, %)	1 (0.3%)	0	0.642
TLR (*N*, %)	0	0	N/A
TVR (*N*, %)	2 (0.6%)	1 (1.4%)	0.479
30-day			
MACE (*N*, %)	30 (8.9%)	11 (15.1%)	0.111
CV MACE (*N*, %)	23 (6.8%)	7 (9.6%)	0.411
Death (*N*, %)	24 (7.1%)	9 (12.3%)	0.138
CV death (*N*, %)	16 (4.7%)	5 (6.8%)	0.460
Fatal MI (*N*, %)	1 (0.3%)	1 (1.4%)	0.233
Nonfatal MI (*N*, %)	3 (0.9%)	0	0.418
Stent thrombosis (*N*, %)	3 (0.9%)	0	0.418
Stroke (*N*, %)	2 (0.6%)	0	0.509
TLR (*N*, %)	2 (0.6%)	1 (1.4%)	0.480
TVR (*N*, %)	4 (1.2%)	2 (2.7%)	0.317
90-day			
MACE (*N*, %)	47 (13.9%)	16 (21.9%)	0.089
CV MACE (*N*, %)	34 (10.1%)	11 (15.1%)	0.221
Death (*N*, %)	32 (9.5%)	10 (13.7%)	0.287
CV death (*N*, %)	18 (5.3%)	5 (6.8%)	0.616
Fatal MI (*N*, %)	1 (0.3%)	1 (1.4%)	0.234
Nonfatal MI (*N*, %)	5 (1.5%)	1 (1.4%)	0.939
Stent thrombosis (*N*, %)	3 (0.9%)	0	0.418
Stroke (*N*, %)	2 (0.6%)	0	0.509
TLR (*N*, %)	9 (2.7%)	4 (5.5%)	0.216
TVR (*N*, %)	12 (3.6%)	6 (8.2%)	0.079
180-day			
MACE (*N*, %)	73 (21.6%)	27 (37.0%)	**0.006**
CV MACE (*N*, %)	54 (16.0%)	21 (28.8%)	**0.011**
Death (*N*, %)	47 (13.9%)	12 (16.4%)	0.589
CV death (*N*, %)	25 (7.4%)	5 (6.8%)	0.861
Fatal MI (*N*, %)	2 (0.6%)	2 (2.7%)	0.091
Nonfatal MI (*N*, %)	8 (2.4%)	2 (2.7%)	0.857
Stent thrombosis (*N*, %)	3 (0.9%)	0	0.418
Stroke (*N*, %)	3 (0.9%)	0	0.418
TLR (*N*, %)	21 (6.2%)	13 (17.8%)	**0.001**
TVR (*N*, %)	26 (7.7%)	15 (20.5%)	0.001
1-year			
MACE (*N*, %)	96 (28.4%)	35 (47.9%)	**0.001**
CV MACE (*N*, %)	65 (19.2%)	27 (37.0%)	**0.001**
Death (*N*, %)	65 (19.2%)	18 (24.7%)	0.306
CV death (*N*, %)	26 (7.7%)	7 (9.6%)	0.599
Fatal MI (*N*, %)	4 (1.2%)	4 (5.5%)	**0.016**
Nonfatal MI (*N*, %)	10 (3.0%)	3 (4.1%)	0.617
Stent thrombosis (*N*, %)	4 (1.2%)	0	0.349
Stroke (*N*, %)	3 (0.9%)	1 (1.4%)	0.707
TLR (*N*, %)	31 (9.2%)	17 (23.3%)	**0.001**
TVR (*N*, %)	37 (10.9%)	20 (27.4%)	**<0.001**

CR, creatinine; CV, cardiovascular; ESRD, end-stage renal disease; MACE, major adverse cardiovascular event; MI, myocardial infarction; TLR, target lesion revascularization; TVR, target vessel revascularization.

**Table 5:** Independent predicting factors for clinical outcomes in multivariable binary logistic regression analysis.

Variables	In-hospital MACE			6-month MACE		
	OR	95% CI	*p* value	OR	95% CI	*p* value
Age	1.12	1.05–1.19	0.001	1.02	1.00–1.05	0.098
Male sex	0.62	0.24–1.63	0.334	1.08	0.64–1.84	0.771
HTN	0.43	0.16–1.14	0.090	0.56	0.33–0.96	0.036
DM	1.11	0.42–2.95	0.836	1.19	0.70–2.02	0.515
PAD	4.42	1.39–14.02	0.012	2.16	1.02–4.57	0.043
Diagnosis of ACS	14.47	2.85–73.42	0.001	4.42	2.02–9.64	**<0.001**
SVD						
DVD			0.996	1.83	0.75–4.43	0.183
TVD + LM			0.996	3.82	1.55–9.45	**0.004**
Cr ≥5 mg/dl through ESRD	5.44	1.74–17.05	0.004	2.36	1.24–4.50	0.009
Ischemic CM/shock	6.85	1.70–27.68	0.007	4.35	1.87–10.10	**0.001**
Hemodynamic support	2.54	0.75–8.60	0.134	1.70	0.85–3.40	0.133
SYNTAX score baseline	0.98	0.94–1.03	0.479	0.99	0.96–1.01	0.307
SYNTAX score post	1.07	1.02–1.12	0.010	1.01	0.98–1.04	0.708

Covariates included in the model were age, sex, HTN, DM, PAD, diagnosis of ACS, CAD vessel numbers, creatinine, ischemic cardiomyopathy/cardiogenic shock, hemodynamic support, SYNTAX score baseline, and SYNTAX score post. OR, odds ratio; 95% CI, 95% confidence interval. ACS, acute coronary syndrome; CM, cardiomyopathy; DM, diabetes mellitus; DVD, double-vessel disease; HTN, hypertension; PAD, peripheral artery disease; SVD, single-vessel disease; TVD, triple-vessel disease.

## Data Availability

The data used to support the findings of this study are available from the corresponding author upon request.
